# Comparison of microleakage in restorative materials used for deep cervical margin elevation: a systematic review

**DOI:** 10.2340/biid.v13.46289

**Published:** 2026-07-01

**Authors:** Shalonny Brithany Garzón Idrovo, Jaqueline Michelle Naula Farez, Martha Alejandra Cornejo Cordova, Maria de Lourdes Leon Vintimilla

**Affiliations:** a Faculty of Dentistry, University of Cuenca, Cuenca, Ecuador

**Keywords:** cervical margin relocation, class II cavity, close sandwich technique, deep marginal elevation, marginal quality, marginal seal, microleakage, open sandwich technique, proximal box elevation

## Abstract

The present study aims to evaluate and compare the rate of microleakage (ML) in deep margin elevation (DME) of posterior teeth with subgingival margins using different materials and techniques. Methodology: An electronic search was conducted between May and August 2025 across three databases, PubMed/Medline, Scopus, and Web of Science, using the following keywords: “deep marginal elevation” OR “proximal box elevation” OR “Cervical margin relocation” OR “coronal margin relocation” OR “open sandwich technique” AND “microleakage.” A secondary search was conducted in PubMed using the same terms without quotation marks. The Preferred Reporting Items for Systematic Reviews and Meta-Analyses (PRISMA) guidelines were applied, and the search was restricted to full-text, in vitro studies published in English between 2014 and 2025 that evaluated ML in DME. Methodological quality was assessed using the Quality Assessment Tool for In Vitro Studies (QUIN) tool by two independent reviewers. Results: A total of 53 articles were screened, and 18 were included. The most frequently compared material among the studies was resin composite, particularly bulk-fill flowable resin composite (BF-FRC) composites, while various types of glass ionomers were less commonly evaluated. Lower ML was observed with the snowplow and centripetal techniques. The type of adhesive was found to influence the marginal integrity of restorations. Conclusion: The evidence highlights the superiority of BF-FRC composites in reducing ML rates in DME when used with self-etch adhesives. Nevertheless, clinicians should remain cautious considering the limitations of the existing evidence. Regarding the technique employed, current evidence is insufficient to determine which approach more effectively reduces ML due to methodological heterogeneity and the lack of direct comparisons.

## Introduction

Interproximal subgingival lesions in posterior teeth represent a frequent challenge in dental practice. Nedeljkovic et al. reported that the prevalence of secondary caries was significantly higher in Black’s Class II cavities, particularly in the gingival area, compared with other cavity classes. Subgingival margins pose a challenge in clinical practice due to their difficult access, instability of the rubber dam, contamination with substances from the oral cavity (saliva, crevicular fluid, and blood) [1], difficulties in taking impressions or intraoral scans, cementation of indirect restorations [2], and the risk of violating the biological width [3].

Several treatment techniques have been proposed to address this issue. Conventional treatment includes orthodontic extrusion, crown lengthening, or a combination of both [4]. However, orthodontic extrusion requires a longer treatment time and increases treatment costs [5]. In contrast, the surgical alternative involves an invasive procedure that may cause greater attachment loss, exposure of root concavities and furcations to the oral environment, dentin hypersensitivity, an unfavorable crown–root ratio, and postoperative discomfort [6].

For this reason, Dietschi and Spreafico first proposed deep margin elevation (DME) in 1998 [7]. This is a minimally invasive procedure that elevates a deep subgingival dental margin to a supragingival level using restorative materials, thereby improving marginal resistance and the integrity of restorations [8] and facilitating restorative procedures [9].

One of the most challenging factors in restorative dentistry is microleakage (ML). According to Kidd, ML is defined as the “clinically undetectable passage of bacteria, fluids, molecules, or ions between a cavity wall and the restorative material applied to it” [10]. Other closely related factors include marginal integrity and marginal adaptation; the difference lies in the fact that the former refers to the precision of the fit at the time the material is placed, whereas marginal adaptation refers to the clinical condition of the marginal interface over time. The main factors influencing the marginal adaptation of restorations are the choice of restorative material, the adhesive system, and the technique used to place the material during DME [11, 12].

Among the materials most commonly used for DME are different types of composite resins, such as conventional composite (CC), bulk-fill resin composite (BFRC), bulk-fill flowable resin composite (BF-FRC), and flowable resin composites (FRC). Additionally, resin-modified glass ionomer cements (RMGIC) and, to a lesser extent, conventional glass ionomer cement (GIC) have also been used. Although these are the materials most frequently reported in the literature for this technique, a wide variety of other restorative materials have also been investigated to a lesser extent.

**Figure 1:**
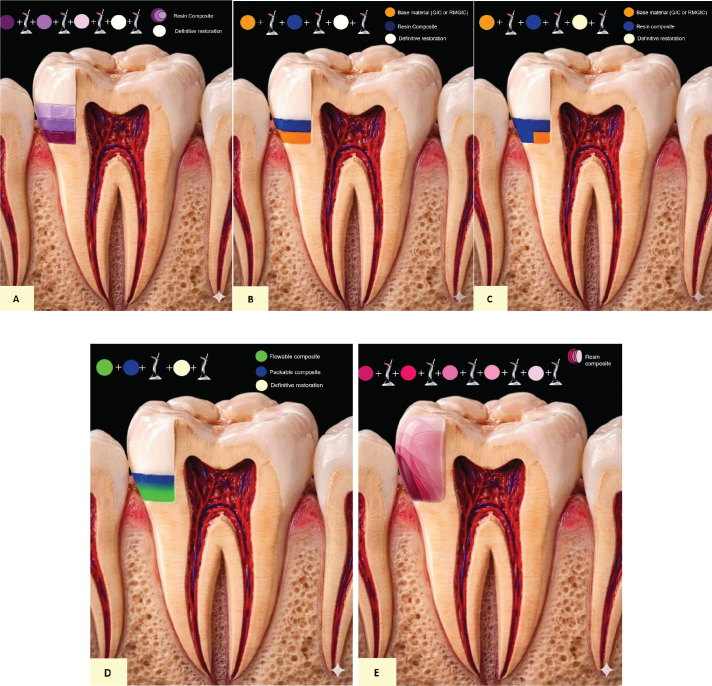
Techniques for deep margin elevation: (A) Incremental technique; (B) Open sandwich technique; (C) Closed sandwich technique; (D) Snowplow technique; (E) Centripetal technique. Illustrations generated using artificial intelligence (Gemini, Google) and adapted by the authors.

Furthermore, scientific research has described different techniques for performing DME. One of the most prominent is the incremental technique, based on the application of three consecutive layers, each with a thickness of 1 mm, as suggested by Roggendorf et al. [13] (Figure 1A). The open and closed sandwich techniques proposed by McLean [14] have also been described. These techniques involve replacing the missing dentin with GIC, while the missing enamel is replaced with composite resins. The difference between the open and closed sandwich techniques is that the former refers to the exposure of part of the GIC or RMGIC to the oral cavity [15], whereas in the closed sandwich technique, the material is placed at the base of the proximal cavity without reaching the external cavosurface margin [16] (Figure 1B and C). Another technique is the snowplow technique, which involves placing a flowable composite at the base of the cavity and subsequently adding a more viscous composite without prior light curing; the materials are adapted and then light-cured together [17], allowing the denser superficial material to compact the deeper viscous material [18] (Figure 1D). On the other hand, the centripetal technique proposed by Hassan et al. [19] replaces the affected dental structure from the periphery toward the center of the cavity by placing a thin proximal layer of material against the matrix band; this technique can also be combined with the open or closed sandwich techniques [20] Figure 1E.

The selection of the appropriate material and technique to minimize ML in DME procedures remains a clinical challenge, as the available evidence does not clearly establish which option ensures optimal marginal sealing [6]. This knowledge gap highlights the need to investigate and compare different restorative approaches. Therefore, this study aims to compare ML in DME performed on posterior teeth with subgingival Class II margins using different materials, techniques, and adhesive systems in order to determine which material offers the best potential performance.

## Materials and methods

This article corresponds to a qualitative study with a descriptive scope, a deductive approach, and a cross-sectional design. The PRISMA 2020 guidelines for the reporting of systematic reviews were used for the development of this review [21]. No prospective protocol registration was performed because the study was converted from a narrative review to a systematic review during peer review, at which point the identification, screening, and data extraction phases had already been partially completed. This limitation is explicitly acknowledged; however, the methodology was defined a priori and applied systematically.

### Eligibility criteria

A research question was formulated based on the PICO strategy (Population, Intervention, Comparison, and Outcomes) (see Table 1). The main research question was: Does DME performed with different materials, techniques, and adhesive systems in subgingival Class II cavities in posterior teeth, when compared with each other, reduce ML according to in vitro scientific studies published between 2014 and 2025?

**Table 1:** Formulation of the research question according to the PICO strategy.

P	Population	Extracted posterior teeth with subgingival Class II cavities
I	Intervention	Deep margin elevation
C	Comparison	Microleakage in different restorative materials, techniques, and adhesive systems
O	Outcomes	Reduction of microleakage

Source: Prepared by the authors.

PICO: Population, Intervention, Comparison, and Outcomes.

#### Inclusion criteria

- Studies published between 2014 and 2025.
- Studies written in English or that could be translated into English.
- Full-text availability.
- In vitro studies

#### Exclusion criteria

- Studies conducted in animals or humans.
- Opinion articles, clinical case reports, and books.
- Conference abstracts
- Articles published before 2014

### Search strategy

The electronic search was conducted between May and August 2025 by three reviewers (S.G., J.N., and M.C.) using three digital databases (PubMed, Scopus, and Web of Science). The following keywords were used: “deep marginal elevation” OR “proximal box elevation” OR “cervical margin relocation” OR “coronal margin relocation” OR “open sandwich technique” AND ML. Additionally, a second search was performed in PubMed using the same terms without quotation marks Table 2.

**Table 2:** Complete electronic search strategies and search parameters used.

Database	Search strategy	Search date
PubMed	“deep marginal elevation” OR “proximal box elevation” OR “cervical margin relocation” OR “coronal margin relocation” OR “open sandwich technique” AND microleakage	May–August 2025
PubMed (second search)	deep marginal elevation OR proximal box elevation OR cervical margin relocation OR coronal margin relocation OR open sandwich technique AND microleakage	May–August 2025
Scopus	(“deep marginal elevation” OR “proximal box elevation” OR “cervical margin relocation” OR “coronal margin relocation” OR “open sandwich technique”) AND microleakage	May–August 2025
Web of Science	(“deep marginal elevation” OR “proximal box elevation” OR “cervical margin relocation” OR “coronal margin relocation” OR “open sandwich technique”) AND microleakage	May–August 2025

Source: Prepared by the authors.

### Study selection and data collection

Two independent reviewers (S.G. and J.N.) performed the study selection as well as the data extraction. Any discrepancies were resolved through discussion and with the involvement of a third reviewer (M.C.). The articles retrieved from the electronic search were initially exported to an Excel Online spreadsheet (Microsoft 365), which was used for the collaborative analysis of the information. The selection of the literature was conducted in four stages. First, the title and abstract of each article were analyzed to determine its eligibility for full-text review according to the inclusion criteria. In the second stage, the search results were exported to a reference management software (Zotero), where duplicate records were identified and removed. In the third stage, full-text versions of paid-access articles were requested through ResearchGate or via email directly from the corresponding author, and articles for which no response was obtained were excluded. In the fourth stage, the full texts were read in detail, and only those that met all the inclusion criteria were selected Table 3.

**Table 3:** Study selection process and number of studies included in the review.

Process stage	Numbers of studies (*n*)
Records identified in databases	109
Records that met the time criterion (2014–2025)	82
Records after removing duplicates	53
Records eligible according to study type (in vitro)	50
Records screened by title and abstract	23
Records after excluding articles without full-text access	18
Studies included in the systematic review	18

Source: Prepared by the authors.

**Figure 2:**
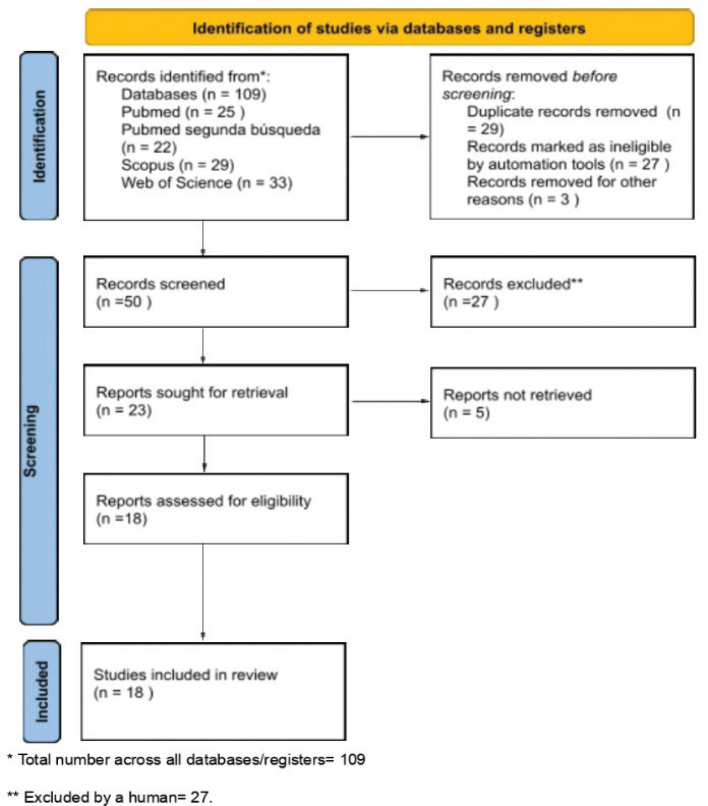
Flow diagram of the study selection process in the systematic review.

Finally, 18 articles were included Figure 2 for the present review, and information such as author, year of publication, study type, and PICO was extracted for comparison and to fulfill the objective of this study Table 4.

**Table 4:** Summary of results from in vitro studies on DME and ML included.

Author, year	Technique	Population	Margin Depth	DME Intervention	Type of restoration	Evaluation method	Results
Juloski J. et al. 2020	Incremental technique	Fourteen intact human molars, healthy and of similar size, without cracks, cavities, or visible restorations.	Mesio-occlusal-distal cavities. On the distal side, no DME was performed, while on the mesial side, DME was performed with a proximal margin located 1 mm below the CEJ.	**Group 1:** Total-etch adhesive system Optibond FL (Kerr) + FCR Premise Flowable (Kerr). **Group 2:** Adhese Universal (Ivoclar Vivadent) with selective enamel etching + Tetric EvoFlow® Bulk Fill (Ivoclar Vivadent).	Hybrid ceramic CAD/CAM blocks Cerasmart, GC (Tokyo, Japan). **Group 1:** Cementation with dual-cure resin cement NX3 Nexus™ (Kerr). **Grupo 2:** Cementation with dual-cure resin cement Variolink Esthetic DC (Ivoclar Vivadent).	ML: Diluted ammoniacal silver nitrate solution for 24 h. Specimens examined with a digital microscope at 1×, 3× y 6× magnifications (Nikon Shuttle Pix, Nikon, Tokyo, Japan) using the following scale: 0: no microleakage. 1: 0–20% of the interface showing microleakage. 2: 20–40% of the interface showing microleakage. 3: 40–60% of the interface showing microleakage. 4: 60–80% of the interface showing microleakage. 5: 80–100% of the interface showing microleakage. Marginal quality: examined under SEM (JSM 6060 LV, JEOL, Tokyo, Japan) at 50× and 200× with the criteria “continuous margin,” “gap/irregularity,” and “not assessable/artifact.”	Significantly lower ML was observed on distal sides, where DME was performed, compared with mesial sides with DME (*p* = 0.000) in both groups separately. With DME (*p* = 0.000) and without DME (*p* = 0.004), Group 2 showed significantly lower ML scores than Group 1. Marginal quality: No significant difference was observed between Groups 1 and 2 in the percentage of gap-free margins in areas with DME (*p* > 0.05). No statistically significant correlations were found at DME sites between ML scores and the percentage of marginal integrity observed under SEM.
Patil BS. et al. 2020	**Group 1:** Open sandwich technique **Group 2:** Snowplow technique **Group 3:** Oblique incremental technique	Thirty extracted intact molars without caries, restorations, or fissures.	Class II with a proximal box and the proximal margin located 1 mm below the CEJ.	**Group 1:** Beautifil II Flow (FRC) + Beautifil II (CC). **Group 2:** Beautifil II Flow. (FCR) + Beautifil II (CC) **Group 3:** Beautifil II. (CC).	Direct restoration with conventional composite (Beautifil II).	Immersion in 2% methylene blue for 24–37 hours. Evaluation with stereomicroscope (Wild M3C, Heerbrugg, Switzerland) at 25x magnification according to ISO criteria: 0: No dye penetration. 1: Penetration up to half of the cervical wall. 2: Penetration along the entire cervical wall. 3: Penetration along cervical and axial walls.	The snowplow restoration technique showed significantly lower ML, followed by the open sandwich restoration, while the oblique incremental technique presented the highest ML values (*p* = 0.0002).
Taori P. et al. 2022	**Group 1:** Open sandwich technique **Group 2:** Closed sandwich technique	Twenty-six sound mandibular first molars without restorations, caries, or cracks were extracted due to periodontal problems.	Mesio-occlusal cavity with the proximal margin 1 mm below the CEJ.	**Group 1:** 1 mm zirconomer base (Zirconomer, Shofu, Japan) **Group 2:** B1 mm zirconomer base (Zirconomer, Shofu, Japan) was left covered within the preparation.	Direct restoration with CC (Dentsply Spectrum Composite) after 37% phosphoric acid etching and dentin adhesive application.	Immersion in methylene blue for 24 h, evaluation with a stereomicroscope (32×; Lobovision, India) and ordinal scoring according to the Pontes DG system: 0: No microleakage. 1: Microleakage limited to enamel. 2: Microleakage reaching dentin. 3: Microleakage extending to the root Surface. Evaluation was later complemented with SEM analysis.	Both groups showed some degree of dye penetration, which was higher in the closed sandwich group, although the difference was not statistically significant (*p* > 0.05). SEM analysis revealed adequate marginal adaptation without gaps or interfacial voids in Group 1, while Group 2 presented les satisfactory marginal adaptation.
Bilgra-mi A. et al. 2022	Open sandwich technique	One hundred and twenty intact samples, without cracks or air bubbles.	Artificial cylindrical model (4 × 4 mm) simulating a Class II restoration, without anatomical reference to the CEJ, fabricated according to ISO 4049 and ISO 10650 standards.	**Group 1:** no DME (control group) **Group 2:** RMGIC (VITREMER) **Group 3:** BF-FRC (Smart Dentin Replacement) **Group 4:** RMGIC (VITREMER) **Group 5:** BF-FRC (Smart Dentin Replacement).	Direct restoration **Group 2:** **A:** Composite resin Z350. **B:** Adhesive (Vitrebond plus or Single Bond Universal, randomly assigned) + Composite resin Z350. **Group 3:** **A:** Composite resin CERAMX **B:** Adhesive (Vitrebond plus or Single Bond Universal, randomly assigned) + Composite resin CERAMX. **Group 4:** **A:** Composite resin CERAMX **B:** Adhesive (Vitrebond plus or Single Bond Universal, randomly assigned) + Composite resin CERAMX. **Group 5:** **A:** Composite resin Z350. **B:** Adhesive (Vitrebond plus or Single Bond Universal, randomly assigned) + Composite resin Z350.	Immersion in 2% methylene for 24 h, evaluation with a stereomicroscope (1040×) and scoring using the WHO Microleakage Index, according to ISO/TS 11405:2003 with a 0–4 scale: 0: 0 mm; 1 ≤ 0.5 mm; 2 ≤ 1 mm; 3 ≤ 2 mm; 4 ≥ 2 mm)	After thermocycling group 2 shoed the highest ML values, with statistically significant differences compared to the other groups (p ≤ 0.05). In contrast, groups 3 and 5 presented minimal or no ML even after thermal aging, suggesting better marginal stability. The BF-FRC Smart Dentin Replacement was used as a base material in the groups where microleakage was observed, indicating better bonding capability with the composite resin material. Conversely, significant ML scores were observed in groups 2 and 4 in this study. The bond between RMGIC Vitremer and the composite resin was weaker, which resulted in higher microleakage values.
Zhang H. et al. 2021	Incremental technique	Eighty human premolars, intact and caries-free, extracted due to dental treatment requirements.	Standardize mesio-occlusal Class II cavities. **Group 1, 2, and 3:** proximal margin 2 mm below the cementoenamel junction **CEJ**. **Group 4:** Proximal margin 1 mm below the CEJ.	Bulk-fill flowable resin composite (BF-FRC) Smart Dentin Replacement (Dentsply Caulk, Milford, DE, USA) with 3 mm thickness. **Group 2:** Conventional composite resin (CC) Filtek Z350 XT (3M ESPE, St Paul, MN, USA) applied in two increments of 1.5 mm. **Group 3:** Cervical margin without DME, located 2 mm apical to the CEJ. **Grupo 4:** Cervical margin without DME, located 1 mm below the CEJ.	Indirect end crown restorations fabricated using a CAD/CAM lithium disilicate reinforced ceramic system (IPS e.max CAD; Ivoclar Vivadent, Schaan, Liechtenstein).	Dye penetration using 0.55% methylene blue (Sigma-Aldrich) for 24 h at room temperature. Observation was performed using a stereomicroscope (SZ51/61, Olympus). Evaluation was carried out at the gingival margin level using an ordinal scoring system (0–4) according to dye penetration depth: 0: no penetration 1: penetration limited to enamel 2: penetration beyond the CEJ up to 2/3 of the cervical wall	The highest frequency of ML scores 0 and 1 was detected in supragingival group 4 (60%), while the highest percentage of score 4 (12%) was found in group 3 (*p* = 0.026). Group 4 also showed the lowest percentage of score 1 (25%, *p* = 0.010), whereas group 1 exhibited a slightly higher frequency of score 2 (*p* = 0.078).
				In all groups, selective enamel etching with 37% orthophosphoric acid (Monobond Plus, Ivoclar Vivadent, Schaan, Liechtenstein) was performed, followed by the application of a self-etch adhesive system (Tetric N-Bond, Ivoclar Vivadent) on the dentin surface.		3: penetration beyond 2/3 of the cervical wall without reaching the pulp 4: penetration up to the pulpal wall	
Aljam-han AS. et al. 2021	**Group 1, 2 y 3:** Incremental technique **Group 4:** Open sandwich technique	Forty recently extracted intact human premolars, confirmed to be free of caries, restorations, cracks, stains, or previous endodontic treatment.	Class II cavities prepared on mesial and distal surfaces, with the proximal margin located 1 mm below the CEJ.	**Group 1:** **A:** Total adhesive etching system (Etch-Rite™ or Optibond™ S) [SG4.1] + CC Tetric N-Ceram (Ivoclar Vivadent, Schaan, Liechtenstein). **B:** OptiBond™ XTR + CC Tetric N-Ceram self-etching adhesive system (Ivoclar Vivadent, Schaan, Liechtenstein). **Grupo 2:** **A:** total adhesive etching system (Etch-Rite™ or Optibond™ S) + BFC SonicFill™ 2 (Kerr Corp., Orange, CA, USA) **B:** OptiBond™ XTR + BFC SonicFill™ 2 self-etching adhesive system (Kerr Corp., Orange, CA, USA) **Grupo 3:** **A:** total adhesive etching system (Etch-Rite™ or Optibond™ S) + Tetric N-Flow FCR liner (Ivoclar Vivadent, Schaan, Liechtenstein) + Tetric N-Ceram CC layer (Ivoclar Vivadent, Schaan, Liechtenstein). **B:** OptiBond™ XTR self-etching adhesive system + Tetric N-Flow FCR liner (Ivoclar Vivadent, Schaan, Liechtenstein). **Grupo 4:** RMGIC Fuji II LC (GC Corporation, Tokyo, Japan) + CC Tetric N-Ceram (Ivoclar Vivadent, Schaan, Liechtenstein).	Direct restoration, not specified in the article or material used.	Dye penetration using 0.5% methylene blue for 24 h, followed by evaluation with a stereomicroscope (×50 magnification). Microleakage was assessed using an ordinal dye penetration scale: 0: no penetration 1: penetration less than half of the gingival wall. 2: penetration up to half of the gingival wall. 3: penetration beyond half of the gingival wall. 4: penetration along the entire gingival wall and axial wall.	The RMGIC group showed the lowest ML score among all groups. No significant difference was observed between the RMGIC group and the BFC group when used with a self-etch adhesive system (*p* = 0.39). The addition of a flowable composite liner under a conventional composite in group 3 reduced microleakage compared with composite without a liner, although the difference was not statistically significant (*p* = 0.59). The total-etch adhesive system showed significantly higher ML compared with the self-etch system in all groups (*p* = 0.026).
Roider M. et al. 2025	Incremental technique	Sixteen human molars and 48 human premolars.	Standardized proximal cavities with mesial and distal defects. **Proximal side with DME:** proximal margin 1 mm below the cementoenamel junction (CEJ). **Control proximal side:** margin located just apical to the CEJ.	**Grupo A y B:** **Control side:** Direct reconstruction with dual-cure composite resin (Clearfil DC Core Plus). **DME side:** A single increment of dual-cure composite resin (Clearfil DC Core Plus). In both cases, a two-step self-etch adhesive system (Clearfil SE Bond 2) was used.	Fixed 3Y-TZP zirconia ceramic prostheses (Katana Zirconia HTML, lot number: EBJED; Kuraray Noritake Dental Inc[SG4.1]) manufactured by CAD/CAM, cemented with resinous cement (Panavia V5, Kuraray Noritake Dental Inc). **Group A:** FDPs with cantilever. **Grupo B:** Conventional FDPs	Penetration of 0.5% fuchsin dye over 24 hours. Evaluation of dye penetration in sectioned sections using a stereomicroscope (Wild M420) at 25× magnification. 0: No leakage 1: Leakage to the vertical level of the cementation start. 2: Leakage to 1/3 of the cervical horizontal shoulder. 3: Leakage to 2/3 of the cervical horizontal shoulder. 4: Leakage to the full length of the cervical horizontal shoulder. 5: Leakage to 1/3 of the axial wall. 6: Leakage to 2/3 of the axial wall. 7: Leakage to the full length of the axial wall. 8: Leakage to 1/3 of the chamfer. 9: Leakage to 2/3 of the chamfer. 10: Leakage to the full length of the chamfer. 11: Leakage up to 1/3 of the occlusal surface. 12: Leakage up to 2/3 of the occlusal surface. 13: Leakage along the entire length of the occlusal surface. 14: Continuous leakage.	All specimens in all groups showed microleakage (ML) at the DME site, specifically at the interface between the tooth and the base restoration. On the control side, a lower incidence of ML was detected. In 84.4% of the control specimens, ML was observed at the interface between the tooth and the resin cement, followed by ML along the interface between the tooth and the base restoration. Higher mean ML values were observed on the DME sides compared with the control sides (*p* < 0.05).
Gyana-ni HC. et al. 2016	Open sandwich technique.	Forty intact human molars recently extracted for therapeutic reasons.	Class II cavities with the proximal margin located 1 mm below the CEJ.	**Group A (RMGIC):** Dentin conditioner + RMGIC Fuji II LC (GC, Japan) applied in 1-mm increments. **Group B (RMGIC):** Same protocol as Group A, with ultrasonic activation of RMGIC. **Group C (Giomer):** Two layers of adhesive (Beautibond®, Shofu Inc., Kyoto, Japan) + Beautifil Flow Plus F03® (Shofu Inc.) applied in 1 mm increments. **Group D (Giomer):** Same protocol as Group C, with ultrasonic activation of the Giomer material.	Direct restoration with Beautifil II using an incremental technique, previously using an adhesive agent (Beautibond®, Shofu Inc., Kyoto, Japan).	Dye penetration in 0.5% methylene blue for 24 h. Observation with a stereomicroscope (Motic, Hong Kong) at 40× magnification. Dye penetration was evaluated according to the criteria of Loguercio et al.: 0: no penetration. 1: up to ½ of the gingival floor. 2: > ½ of the gingival floor. 3: up to the axial wall.	Group B (RMGIC with ultrasonic activation) showed the best results. In Groups B and D, ultrasonic activation accelerated the chemical curing process, increasing the surface area of the powder due to the breakdown of glass particles by ultrasonic energy, which increased reactivity and improved physical properties. The results showed that ultrasonic groups tended to present lower ML values. Group C (Giomer) showed better performance than Group A (RMGIC).
Feiz A. et al. 2021	**Group 1:** Open sandwich technique **Group 2, 3 y 4:** Incremental technique	Sixty extracted human third molars, free of cracks, fractures, caries, abrasion, previous restorations, or structural defects.	Class II cavities prepared on mesial and distal surfaces, with the proximal margin located 1 mm below the CEJ.	**Group 1:** 10% polyacrylic acid conditioning + 1-mm RMGIC base (Fuji II LC). **Group 2:** 37% phosphoric acid etching of enamel + adhesive system (Clearfil SE Bond) + 1-mm RMGIC (Ionoseal). **Group 3:** Acid etching + adhesive system (same as group 2) + 1-mm conventional composite (x-tra base). **Group 4:** Acid etching + adhesive system (same as group 2) + 1-mm flowable composite resin (Grandio Flow) placed as the base of the cavity preparation. **Group 5 (control):** No DME.	Direct restoration using two layers of bulk-fill composite (x-tra fil) of 3 and 4 mm thickness. Only in **Group 1:** enamel etching with 37% phosphoric acid + Clearfil SE Bond adhesive before restoration.	Immersion in 0.5% basic fuchsin for 24 hours. Observation of the cavity preparation base using a stereomicroscope (32×). The greatest dye penetration in each half was recorded. Evaluation was performed using a 4-point scale according to ISO/TS 11405:2003: 0: no penetration. 1: penetration to the mid-gingival floor. 2: penetration beyond the mid-gingival floor without reaching the axial wall. 3: penetration to the axial wall.	The highest dye penetration occurred in the Ionoseal (RMGIC) group and the control group, with no significant differences between them. Dye penetration in the other three groups was significantly lower than in the control and Ionoseal groups (*p* < 0.02). Except for the Ionoseal group, all other groups showed significantly lower ML than the control group (*p* < 0.02); however, no significant differences were observed among these groups (*p* > 0.05).
Reddy. et al. 2024	Incremental technique	Thirty recently extracted human maxillary molars removed for periodontal reasons, without caries or fractures.	Class II cavities with the proximal margin 1 mm below the CEJ.	**Group A:** 37% orthophosphoric acid + adhesive (Adper Single Bond 2, 3M ESPE) + 1-mm layer of FCR (GrandioSO Heavy Flow, VOCO). **Group B:** 1 mm layer of GIC (GC Gold Label, GC Corporation, Tokyo, Japan) placed on the gingival floor. **Group C:** 1 mm base layer of GIC with nano-hydroxyapatite (Nanowings, sol-gel method, Pvt. Ltd., HYD).	Direct restoration with BFC (3M™ Filtek™ One Bulk Fill Restorative). Only in Groups B and C: 37% orthophosphoric acid + adhesive (Adper Single Bond 2, 3M ESPE) before final restoration.	Samples were immersed in 0.5% rhodamine B for 2 days; sections were evaluated using a Stellaris 5 confocal microscope in a standardized 273 × 273 µm area at depths of 211 µm and 273 µm. Dye penetration was evaluated on a scale of 0–3: 0: no penetration 1: up to half of the cervical wall 2: more than half 3: from the cervical wall to the axial/progress toward the pulp. The integrity of the interface between the base material and the bulk filling composite was examined using high-resolution JSM-IT800 NANO SEM, observing the critical interface at magnifications from ×25 to ×500.	A significant difference in mean ML values was observed. The nano-hydroxyapatite GIC group showed the lowest mean ML (1.4) compared with flowable composite resin (2.2) and conventional GIC (2.8). SEM analysis revealed that although the nano-hydroxyapatite GIC group showed better ML performance, it exhibited a greater mean gap distance (17.384 µm) compared with the FCR (11.276 µm) and GIC (12.116 µm) groups.
Senol. et al. 2023	Incremental technique	Sixty extracted human mandibular molars, non-carious, non-cracked, and with similar dimensions.	Mesio-occluso-distal cavities with cervical margins 1 mm below the CEJ on one side and 1 mm above the CEJ on the other.	**Group 1:** X-tra Base Voco BF-FCR, 4 mm increment at gingival floor **Group 2:** Same material, 2 mm increment **Group 3:** Tetric N-Flow Bulk Fill (Ivoclar Vivadent), 4 mm increment **Group 4:** Same material, 2 mm increment **Group 5:** Admira Fusion Base Ormocer (flowable), 4 mm increment **Group 6:** Same material, 2 mm increment	**Group 1:** Voco X-tra Fil high-load resin, 2 mm increments. **Group 2:** Same as Group 1, in 4 mm increments. **Group 3:** Ivoclar Vivadent Tetric N Body high-viscosity resin, 2 mm increments. **Group 4:** Same as Group 3, in 4 mm increments. **Group 5:** Voco Admira Fusion X-tra bulk-fill resin, Ormocer, 2 mm increments. **Group 6:** Same as Group 5, in 4 mm increments.	Dye penetration was assessed using 0.2% methylene blue solution for 24 hours. Gingival and coronal microleakage was evaluated using a stereomicroscope (Leica MZ75) and SEM (FEI Sirion-10kV). Gingival microleakage was scored from 0 to 3: 0: no penetration 1: up to half the cervical wall 2: more than half 3: cervical and axial penetration to the pulp. Subsequently, samples from each group with a gingival microleakage score of 2 or 3 were selected and micrographed using SEM. The samples were examined at ×120, ×500, and ×5000 magnifications.	No statistically significant difference was found in the distribution of ML at the DME–cementum interface between groups 1 and 2, and between groups 5 and 6 (*p* = 0.604 and *p* = 0.481, respectively). However, group 3 showed higher ML values with a score of 3 compared to group 4 (*p* = 0.018). Furthermore, no statistically significant difference was detected in the DME–enamel and DME–cementum ML between groups 1, 3, and 5 (*p* = 0.112 and *p* = 0.563, respectively).
Çelik Ç. et al. 2020	Open sandwich technique	Sixty extracted human molars without caries, cracks, or restorations.	Standardized Class II cavities were prepared with the gingival margin located 1 mm below	**Group I:** High-viscosity GIC (Fuji IX GP) 1 mm thick. **Group II:** RMGIC (Fuji II LC) 1 mm thick. **Group III:** Hybrid glass ionomer (Equia-fil Forte) 1 mm thick. **Group IV (control):** Without DME.	Direct restoration with CC (G’aenial Posterior, GC, Tokyo, Japan) previously applied universal adhesive (GPremio Bond, GC, Tokyo, Japan)	The distal cavities of all restorative material groups were contaminated with saliva for 5 seconds using a microbrush. Immersion in 0.5% methylene blue was performed for 24 hours. Penetration was assessed at 20X magnification using a stereomicroscope (Nexius Zoom, Euromex Microscopen BV, Arnheim, The Netherlands). The degree of dye penetration was quantitatively evaluated using image analysis software (Imagen J, V.1.42, National Institutes of Health, Bethesda, MD).	Under contamination-free conditions, the DME with Fuji IX GP glass ionomers showed greater ML (*p* < 0.05) than the groups with RMGIC (Fuji II LC and Equia-fil Forte) and the control. Under saliva contamination, there were no significant differences between the groups (*p* > 0.05). In the absence of contamination, hybrid glass ionomers and RMGIC showed lower ML at the gingival margins than high-viscosity ionomers, making them preferable when isolation is adequate.
Lorca. et al. 2023	Incremental technique	Forty-eight healthy human molars, with an indication for surgical or orthodontic extraction	Mesio-occlusal and disto-occlusal cavities, with the proximal margin 2 mm below the CEJ	**Group A:** CC (Filtek™ Z250 3M ESPE) in increments less than 2.5 mm thick. **Group B:** BF-FCR (Filtek™ Bulk Fill, 3M ESPE) single-increment technique up to 5 mm thick.	Semi-direct inlays placed over the DME in each molar using CC Filtek™ Z350™ A2 Body, and cemented with RelyX™ U200™ (3M ESPE).	The specimens were immersed in a 50% colloidal silver nitrate solution for 24 hours. Dye penetration was analyzed using a stereoscopic magnifying glass at 40× magnification and a translucent millimeter grid to establish measurements at 40×.	No significant differences were found in lateral ML between BF-FCR (Filtek™ Bulk Fill) and CC (Filtek™ Z250) when using DME (*p* = 0.6848), although BF-FCR showed a trend toward lower ML (28.6%) compared with 33.1% in the conventional group.
						A digital photograph of each restoration was taken as a record for analysis using the color-analysis software ImageJ, and the percentage of infiltrated area was calculated.	Both materials are suitable for cavities located below the CEJ.
Koken. et al. 2019	Incremental technique	Twenty intact, healthy human third molars of similar size, extracted for therapeutic purposes, without cracks, caries, or visible restorations.	Mesio-occluso-distal Class II cavities with the proximal margin 1 mm below the CEJ. **Mesial surfaces:** with DME **Distal surfaces:** without DME	**Group 1 and 2:** **Mesial side:** DME with universal adhesive (G-Premio Bond, GC Corp.) + two increments of 1 mm each of Gænial Universal Flo (GC Corp., Tokyo, Japan). **Distal side:** without DME	Cerasmart overlays (GC Corp.) cemented with resin cement (GCem LinkForce, GC Corp.). **Adhesive system for cementation** **Group 1:** adhesive system G-Premio Bond (GC Corp.) with selective enamel etching. **Group 2:** three-step total-etch adhesive system (Optibond FL, Kerr, Orange, CA, USA).	Dilute ammoniacal silver nitrate solution for 24 h. They were examined with a digital microscope at 1x, 3x, and 6x magnification (Nikon Shuttle Pix, Tokyo, Japan) with the following scoring: 0: no microleakage. 1: 0–20% of the interface shows microleakage. 2: 20–40% of the interface shows microleakage. 3: 40–60% of the interface shows microleakage. 4: 60–80% of the interface shows microleakage.	Group 1 (cemented with G-Premio Bond) showed significantly higher ML in areas with DME than without DME (*p* = 0.000). In Group 2 (cemented with OptiBond FL), no significant differences were found between areas with and without DME (*p* = 0.491). Furthermore, when comparing sites without DME between groups, G-Premio Bond showed significantly lower ML than OptiBond FL. The type of adhesive used for cementation significantly influenced ML below the CEJ when DME was not performed. The universal adhesive showed better results than the three-step system under these conditions.
Guior-ghe. et al. 2017	Open sandwich technique	Thirty molars extracted for orthodontic reasons.	Proximal Class II cavities with the proximal margin 1.5 mm below the CEJ.	**Control group:** No DME. **Group 1 subdivided into:** **1 F-D =** Dyract compomer **1 F-KM =** Ketac Molar Easymix GIC **Group 2 subdivided into:** **2 Z-D =** Dyract compomer. **2 Z-KM =** Ketac Molar GIC.	**Group 1:** Filtek Z250 conventional composite (CC) placed in incremental layers. **Group 2:** Zmack Comp composite placed in incremental layers.	The sections were immersed in a 1% methylene blue solution (Vitalia Pharma, Romania) for 24 h. The sections were examined using digital optical microscopy with a Leica CTR 4000 (Leica Microsystems) and a QUANTA 200 3D SEM microscope (FEI, USA) in ESEM (Environmental SEM) mode. The ML (Methylase) was evaluated using a scoring system from 0 to 3 according to ISO/TS 11405-2003: 0: no dye penetration. 1: dye penetration into the enamel/cementum portion of the cavity wall. 2: dye penetration into the dentin portion of the cavity wall, excluding the pulpal floor of the cavity. 3: dye penetration including the pulpal floor of the cavity.	The lowest ML values corresponded to Group 2 Z-D and the control group, while Group 1 F-KM and Group 2 Z-KM showed higher ML and poorer sealing. Greater dye penetration was observed with GIC compared with compomer using the open sandwich technique, although this difference was statistically significant only when the overlying resin was Filtek Z250. In general, groups restored with Filtek Z250 showed greater ML than those restored with Zmack Comp.
							The best marginal adaptation was achieved with Dyract eXtra compomer and Zmack Comp resin, highlighting the importance of appropriate material selection depending on the clinical situation.
Zavattti-ni. et al. 2018	Instrumental technique	Thirty healthy molars extracted for periodontal reasons, fully developed and without pathology.	Mesio-occluso-distal cavities with the proximal margin 1.5 mm below the CEJ.	**Preheated composite group:** Three-step etching and rinsing adhesive system, OptiBond FL (Kerr) + 1.0–1.5 mm thick layer of composite resin (Premise Dentin A3 Kerr BN. 4396739) (mesial and distal). **FCR group:** Application of the same adhesive system as the previous group + 1.0–1.5 mm thick layer of FCR (Premise flowable Kerr BN. 4264444) (mesial and distal). **Control group:** Restoration without MDF.	Direct restoration using 3–4 layers of unspecified conventional composite, placed with a horizontal layering technique of 1–1.5 mm.	Direct restoration using 3–4 layers of unspecified conventional composite, placed with a horizontal layering technique of 1–1.5 mm. Microleakage evaluation Specimens were immersed in 50% ammonia solution as a tracer for 24 hours. ML analysis was performed using a SkyScan 1072 micro-CT system (SKYSCAN, Kontich, Belgium) with a final resolution of 19.1 µm × 19.1 µm. The ML pattern was digitally evaluated using the TView SkyScan system, allowing visualization of all micro-scans and detection of ammonia leakage. The obtained data were evaluated based on the recorded volume of dye ML expressed in mm³.	Statistically significant differences in ML were observed between enamel and dentin margins. Descriptive statistical analysis showed that FCR exhibited the highest ML, with a mean of 0.74 mm³ at the cementum margin. The use of FCR at the dentin–cementum margin should be avoided; however, when the margin is below the CEJ, the application of preheated composite is recommended to reduce ML incidence.
Raju. et al. 2014	**Group 1:** Incremental technique. **Group 2:** Open sandwich	Seventy human molars, 50 molar teeth were used for microleakage (ML) testing.	Class II occluso-proximal cavities with the proximal margin 2.5 mm below the CEJ.	**Tricalcium silicate restorative material group:** (Biodentine™, Septodont, Saint-Maur-des-Fossés, France), with a thickness of 3 mm. **Posterior radiopaque GIC group:** (Fuji IX GP, GC Corporation, Tokyo, Japan), using the open sandwich technique.	Direct restoration with unspecified conventional composite (CC) using the incremental technique.	The samples were placed in a 5% methylene blue stain for 12 h at room temperature. The degree of stain penetration was studied using a stereomicroscope at 40x magnification with the following criteria: 1: No stain penetration. 2: Partial penetration. 3: Penetration to the gingival wall, but not reaching the axial wall. 4: Penetration to and along the axial wall.	GIC exhibited greater ML than the tricalcium silicate-based restorative material, which was statistically significant in both permanent teeth (*p* = 0.02) and primary teeth (*p* = 0.006). Biodentine showed good marginal integrity and lower ML at the bonding interface between the material and dentin.
Sawani. et al. 2014	**Group 1, 2, 5 and 6:** Centripetal technique with an open sandwich. **Group 3 and 4:** Centripetal technique with a closed sandwich **Group 7:** Centripetal technique	Fifty-three human mandibular molars, non-carious and recently extracted, with approximately similar dimensions.	Standardized Class II cavities on the mesial and distal surfaces with the proximal margin 1 mm below the CEJ.	**Group 1:** 1 mm of FCR (Filtek 350 XT flow, 3M/ESPE, USA) on the gingival seat of the proximal box. **Group 2:** 1 mm RMGIC (Vitrebond, 3M/ESPE, USA) on the gingival seat of the proximal box. **Group 3:** 1 mm of FCR (Filtek 350 XT flow, 3M/ESPE, USA) on the pulp floor and axial wall. **Group 4:** with 1 mm RMGIC (Vitrebond, 3M/ESPE, USA) on the pulp floor and axial wall. **Group 5:** with 1 mm FCR (Filtek 350 XT flow, 3M/ESPE, USA) on the pulp floor, axial wall, and gingival seat. **Group 6:** with 1 mm RMGIC (Vitrebond, 3M/ESPE, USA) on the pulpal floor, axial wall, and gingival margin. **Group 7:** Centripetal mesio- and disto-occlusal technique without any investment material.	Direct restoration with 37% orthophosphoric acid (Scotchbond MultiPurpose, 3M/ESPE, USA) + adhesive (Adper Single Bond 2 Adhesive, 3M/ESPE, USA) + nanocomposite placement (Filtek 350 XT restorative material, 3M/ESPE, USA).	Dye penetration in 0.5% basic fuchsin for 24 h, observed with a stereomicroscope (Carl Zeiss, Italy) at 12×, and evaluated according to the ISO ML system: 0: no penetration 1: half of the cervical wall 2: the entire cervical wall 3: cervical and axial section.	Cervical margin assessments showed that the control group without lining material had the lowest ML scores (*p* < 0.05), followed by Group 4 < Group 3 < Group 1 < Group 5 < Group 6 < Group 2. Among the experimental groups, ML assessments for the closed sandwich technique at the cervical margin were significantly lower than for the open sandwich technique (*p* < 0.05). The FCR used in the open sandwich centripetal reconstruction performed slightly better in terms of ML reduction compared to the RMGIC.

Source: Authors owns elaboration.

DME: Deep margin elevation; ML: Microleakage; SEM: Scanning electron microscopy; FDP: Fixed dental prosthesis; GIC: Glass ionomer cement; RMGIC: Resin-modified glass ionomer cement; CC: Conventional composite; BFC: Mass-fill composite; FCR: Flowable resin composite; BF-FRC: Mass-fill flowable resin composite; h: hours; WHO: World Health Organization; ISO: International Organization for Standardization; CAD/CAM: Computer-Aided Design/Computer-Aided Manufacturing.

### Analytical approach

The included studies were grouped according to the material used for DME, ordered from the most to the least frequently used in the studies. Additionally, the restorative technique evaluated and the adhesive system employed were considered in order to allow a structured comparison between interventions with similar characteristics.

No statistical conversions were performed for the analysis; the data were reported as presented in the original studies. The 18 included studies were systematized in a summary table in Excel Online (Microsoft 365), which compiled their methodological characteristics (author, technique, population, margin depth, intervention, type of restoration, evaluation method, and main results). Subsequently, the findings were integrated through a narrative synthesis of the included studies. No meta-analysis was performed due to substantial heterogeneity among the included studies in terms of experimental design, outcome measures, and the materials and techniques evaluated, which precluded data comparability and quantitative synthesis.

### Methodological quality assessment of the included studies

The methodological quality of the included studies was independently assessed by two investigators (S.G. and J.N.) using the Quality Assessment Tool for In Vitro Studies (QUIN tool), which was specifically developed and validated for in vitro studies in dentistry [22] and demonstrates adequate validity and internal consistency. In cases of disagreement between the reviewers, a third evaluator (M.C.) was consulted to reach consensus. The assessment was based on a predefined set of domains specified in Table 5. Subsequently, each study was classified according to its level of risk of bias as low, moderate, or high, based on the previously defined evaluation criteria. Inter-reviewer agreement for the risk of bias assessment was estimated using Cohen’s kappa coefficient. The GRADE approach was not applied due to its limited validity for in vitro studies, whose methodological characteristics and lack of clinical outcomes may compromise the assessment of the certainty and applicability of the evidence. In addition, it should be noted that GRADE is not specifically designed for in vitro research, as such studies inherently begin with a lower level of evidence, which may lead to an underestimation or misrepresentation of their methodological value. In this context, and considering the inherent limitations of this type of design, the QUIN tool was used to assess the methodological quality of the included studies [23, 24].

**Table 5:** QUIN tool (risk of bias tool for assessing in vitro studies).

Criteria	Details
1. Clearly stated aims/objectives	Study should clearly state aims and/or objectives, which should then be followed throughout.
2. Detailed explanation of sample size calculation	Details regarding method by which given sample size calculation could be clearly stated. Details regarding software program, formula, and parameters used for calculation of sample size also to be specified.
3. Detailed explanation of sampling technique	Details regarding predefined population from which sample has been selected. Details of sampling technique and inclusion and exclusion criteria should be clearly stated.
4. Details of comparison group	Details of comparison group (positive control, negative control, or standard) should be clearly specified.
5. Detailed explanation of methodology	Clarity of procedure, method of standardization, and details of any universal standards used (if applicable) should be clearly stated.
6. Operator details	Number of operators and details regarding training and calibration of operator/s (inter-operator and intra- operator reliability) should be clearly specified.
7. Randomization	Details regarding sequence generation and allocation concealment should be clearly stated.
8. Method of measurement of outcome	Clarity of procedure and rationale for choosing method should be stated. Method of standardization along with details of any universal standards used (if applicable) should be clearly specified.
9. Outcome assessor details	Number of outcome assessors and details regarding training and calibration of assessor/s (inter- outcome and intra-outcome assessor reliability) should be clearly specified.
10. Blinding	Details regarding blinding of operator(s), outcome assessor(s), and statistician should be clearly specified.
11. Statistical analysis	Details regarding software program used and statistical analysis should be clearly specified.
12. Presentation of results	Outcome should be based on predefined aims and/or objectives. All data should be adequately tabulated with baseline data clearly specified (if applicable).

Source: https://pubmed.ncbi.nlm.nih.gov/35752496/.

### Reporting according to PRISMA

The review was conducted and reported in accordance with the PRISMA 2020 statement [21] to ensure transparency and methodological rigor in the identification, selection, evaluation, and synthesis of the included studies. The PRISMA 2020 checklist is presented in Appendix 1.

## Results

### Literature search

Following the search strategy, a total of 109 articles were identified. Twenty-seven articles were excluded because they did not meet the inclusion criteria regarding the publication period (2014–2025). After removing duplicate records, 53 articles remained for screening, of which three were excluded for not meeting the in vitro inclusion criteria. After screening the titles and abstracts, 27 articles were excluded because they were not related to the topic of the present review. For the analysis of the articles, all attempts were made to obtain the full-text versions; however, five articles could not be accessed. Finally, 18 articles that met all the criteria established for this study were included in the present review, as detailed in Figure 2.

### Quality assessment of the included studies

**Figure 3:**
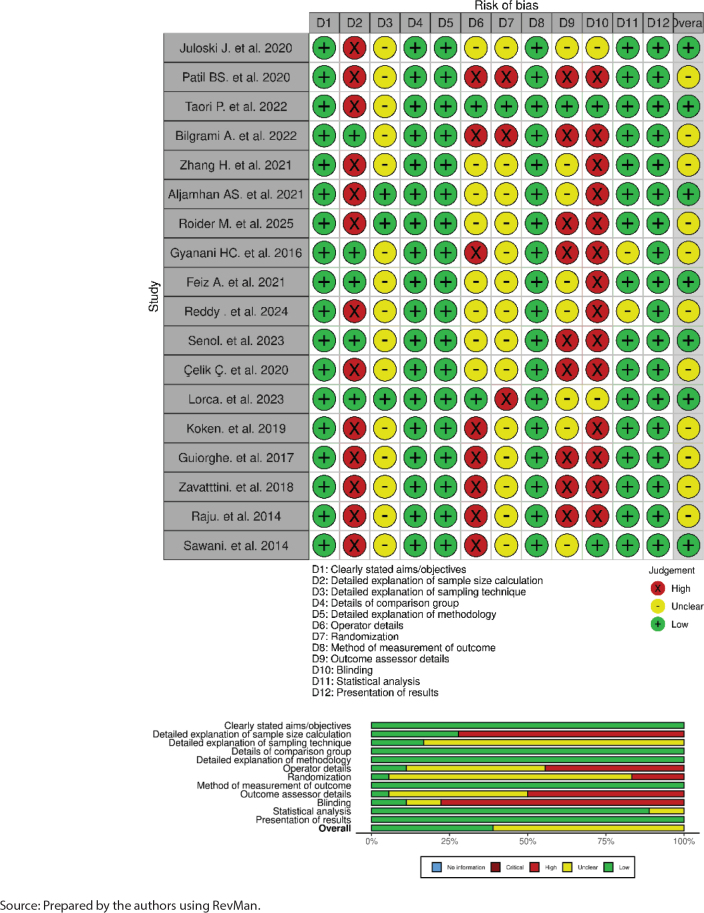
Summary of risk bias.

The studies included in this review showed different levels of risk of bias, ranging from low to moderate, according to the evaluation performed using the QUIN tool. Of the 18 in vitro studies analyzed, only seven achieved a low risk of bias, obtaining scores higher than 70% [9, 16, 25–29]. In contrast, the remaining 11 studies were classified as having a moderate risk of bias, with scores ranging between 50 and 70% [11, 12, 18, 30–37]. Two common findings among the studies were the lack of detailed information regarding the procedures used to explain the sample size calculation, except in five studies [26–28, 30, 32], and the absence of a detailed explanation regarding blinding, except in four studies [9, 16, 28, 29]. The complete results of the quality assessment are presented in Table 6 and Figure 3. Inter-reviewer agreement in the risk of bias assessment was high, with a Cohen’s kappa coefficient of κ = 0.84, indicating a high level of agreement between the reviewers.

**Table 6:** Methodological quality assessment of the included studies according to the QUIN tool.

Study	Criteria 1	Criteria 2	Criteria 3	Criteria 4	Criteria 5	Criteria 6	Criteria 7	Criteria 8	Criteria 9	Criteria 10	Criteria 11	Criteria 12	Total score	Final score %	Risk of bias
Juloski J. et al. 2020	2	0	1	2	2	1	1	2	1	1	2	2	17	70.83	Low
Patil BS. et al. 2020	2	0	1	2	2	0	0	2	0	0	2	2	13	54.17	Medium
Taori P. et al. 2022	2	0	1	2	2	2	2	2	2	2	2	2	21	87.5	Low
Bilgrami A. et al. 2022	2	2	1	2	2	0	0	2	0	0	2	2	15	62.5	Medium
Zhang H. et al. 2021	2	0	1	2	2	1	1	2	1	0	2	2	16	66.7	Medium
Aljamhan AS. et al. 2021	2	0	2	2	2	1	1	2	1	0	2	2	17	70.8	Low
Roider M. et al. 2025	2	0	2	2	2	1	1	2	0	0	2	2	16	66.7	Medium
Gyanani HC. et al. 2016	2	2	1	2	2	0	1	2	0	0	1	2	15	62.5	Medium
Feiz A. et al. 2021	2	2	1	2	2	1	1	2	1	0	2	2	18	75	Low
Reddy et al. 2024	2	0	1	2	2	1	1	2	1	0	1	2	15	62.5	Medium
Senol. et al. 2023	2	2	1	2	2	1	1	2	0	0	2	2	17	70.8	Low
Çelik Ç. et al. 2020	2	0	1	2	2	1	1	2	0	0	2	2	15	62.5	Medium
Lorca. et al. 2023	2	2	2	2	2	2	0	2	1	1	2	2	20	83.3	Low
Koken. et al. 2019	2	0	1	2	2	0	1	2	1	0	2	2	15	62.5	Medium
Guiorghe. et al. 2017	2	0	1	2	2	0	1	2	0	0	2	2	14	58.3	Medium
Zavatttini. et al. 2018	2	0	1	2	2	0	1	2	0	0	2	2	14	58.3	Medium
Raju. et al. 2014	2	0	1	2	2	0	1	2	0	0	2	2	14	58.3	Medium
Sawani. et al. 2014	2	0	1	2	2	0	1	2	1	2	2	2	17	70.8	Low

Source: Prepared by the authors.

Adequately specified = 2; Inadequately specified = 1; Not specified = 0.

Final score: 70% = Low risk of bias; 50–70% = Moderate risk of bias; < 50% = High risk of bias.

Final score = Total score × 100 / (2 × number of applicable criteria).

### Individual study results

Juloski et al. [9] evaluated BF-FRC and FRC and reported that samples with DME using BF-FRC (Tetric EvoFlow® Bulk Fill, Ivoclar Vivadent, Liechtenstein) and a universal adhesive (Adhese® Universal, Ivoclar Vivadent, Liechtenstein) showed significantly lower ML scores compared with those restored with FRC (Premise™ Flowable, Kerr Corporation, Orange, CA, USA) (*p* < 0.001) and a three-step adhesive system (OptiBond™ FL, Kerr Corporation, Orange, CA, USA).

Similarly, Patil et al. [18] analyzed ML in DME using CC and giomer-type FRC with three techniques: open sandwich, snowplow, and oblique incremental techniques. For the open sandwich and snowplow techniques, giomer nanohybrid CC (Beautifil II, Shofu Inc., Kyoto, Japan) was used in combination with giomer FRC (Beautifil II Flow, Shofu Inc., Kyoto, Japan), whereas the oblique incremental technique used exclusively the giomer nanohybrid CC. ML was significantly lower with the snowplow technique compared with the open sandwich technique (*p* = 0.02), which in turn showed lower ML than the oblique incremental technique (*p* = 0.05).

In another study, Taori et al. [16] investigated the open and closed sandwich techniques, using zirconomer (Zirconomer, Shofu Inc., Kyoto, Japan) as the base material for both groups. The results indicated the presence of ML in both groups, although a trend toward higher scores was observed in the closed sandwich technique (*p* = 0.19). Additionally, complementary evaluation using scanning electron microscopy (SEM) supported these observations.

Bilgrami et al. [30] focused their investigation on comparing DME performed using the open sandwich technique with either RMGIC (Vitremer™, 3M ESPE, St. Paul, MN, USA) or BF-FRC (SDR®, Dentsply Sirona, Konstanz, Germany). The difference between the groups was based on the presence or absence of a universal adhesive (Single Bond™ Universal, 3M ESPE, St. Paul, MN, USA) at the interface between the DME layer and the direct restoration. The results showed that both groups using BF-FRC as the base material exhibited null or minimal ML scores compared with the groups using RMGIC (*p* < 0.05).

Zhang et al. [31] reported that the highest ML scores were observed in the group without DME at 2 mm from the cemento-enamel junction (CEJ), whereas the lowest scores were found in the group without DME at 1 mm from the CEJ (*p* = 0.026). Additionally, no significant differences were found between DME samples using BF-FRC (Dentsply Caulk, Dentsply Sirona, Milford, DE, USA) and CC (Filtek™ Z350 XT, 3M Oral Care, St. Paul, MN, USA). Furthermore, fracture resistance was also evaluated, and a significant increase was reported in the groups with DME compared with the control group (*p* = 0.01). Among the DME groups, the highest fracture resistance was observed when BFRC was used; however, no significant differences were found when CC was employed (*p* = 0.07).

Aljamhan et al. [25] evaluated ML in DME using BFRC, FRC, and RMGIC as liners, combined with either etch-and-rinse or self-etch adhesive systems. The results showed that the RMGIC group exhibited the lowest ML scores among all groups. When an etch-and-rinse adhesive system was used, a trend toward lower ML levels was observed with the combination of FRC and CC, respectively, compared with BFRC (*p* = 0.59). However, no significant differences were found between RMGIC and BFRC when a self-etch adhesive system was used (*p* > 0.05).

Roider et al. [11] analyzed ML associated with DME using a CC dual-curing composite core material with both light- and self-curing polymerization. In the DME groups, a dual-curing composite (Clearfil DC Core Plus, Kuraray Noritake Dental Inc., Tokyo, Japan) was used, and the gingival margin of the cavity was located 2 mm below the CEJ, whereas in the groups without DME, it was positioned 1 mm below the CEJ. In both groups, a two-step self-etch adhesive system (Clearfil SE Bond 2, Kuraray Noritake) was applied. The results showed that all samples in the DME groups exhibited greater ML at the tooth–composite core interface, whereas samples without DME showed a significantly lower incidence of ML (*p* < 0.05).

Gyanani et al. [32] evaluated ML after performing DME using the open sandwich technique, comparing the application with and without ultrasonic agitation of the restorative materials. Two materials were tested: RMGIC (Fuji II LC, GC Corporation, Tokyo, Japan) and a giomer-based FRC (Beautifil II Flow, Shofu Inc., Kyoto, Japan). Overall, a trend toward lower ML was observed in the groups with ultrasonic activation, particularly in the RMGIC group. However, these differences were not statistically significant (*p* > 0.05).

Similarly, Feiz et al. [26] investigated the open sandwich technique for DME using different materials: RMGIC (Fuji II LC, GC Corporation, Tokyo, Japan), RMGIC (Ionoseal®, VOCO GmbH, Cuxhaven, Germany), BF-FRC (x-tra base®, VOCO GmbH), and FRC (Grandio® Flow, VOCO GmbH). In addition, a control group without DME, restored with CC (x-tra fil®, VOCO GmbH), was included. The results showed that the control group and the group restored with RMGIC Ionoseal exhibited higher ML compared with the other groups (*p* < 0.02).

Reddy et al. [33] compared ML in DME using FRC (GrandioSO Heavy Flow®, VOCO GmbH, Cuxhaven, Germany), GIC (GC Gold Label, GC Corporation, Tokyo, Japan), and GIC modified with nano-hydroxyapatite (Nanowings Pvt. Ltd., Hyderabad, India). A two-step adhesive system (Adper Single Bond 2, 3M ESPE, St. Paul, MN, USA) was applied in all groups. The results demonstrated that the nano-hydroxyapatite-modified GIC group showed significantly lower ML compared with the FRC (GrandioSO Heavy Flow) and conventional GIC (GC Gold Label) groups (*p* < 0.05). However, SEM analysis revealed that samples restored with nano-hydroxyapatite-modified GIC exhibited a higher mean interfacial gap distance (17.384 µm) compared with the FRC (11.276 µm) and GIC (12.116 µm) groups.

Senol et al. [27] evaluated ML in DME using different BF-FRC: X-tra Base® (VOCO GmbH, Cuxhaven, Germany), Tetric N-Flow Bulk Fill (Ivoclar Vivadent, Schaan, Liechtenstein), and an ormocer-based BF-FRC (Admira Fusion x-base, VOCO GmbH, Cuxhaven, Germany), comparing increment thicknesses of 2 and 4 mm. Overall, the results revealed no significant differences in ML at the tooth–restoration interface among the groups, including the control group. However, when only the DME groups were analyzed, Tetric N-Flow Bulk Fill placed in a 4-mm increment showed significantly higher ML than when applied in a 2-mm increment (*p* = 0.01). The remaining groups did not show significant differences in ML levels at this interface.

On the other hand, Çelik et al. [34] compared GIC (Fuji IX GP, GC Corporation, Tokyo, Japan) and RMGIC (Fuji II LC and Equia Forte®, GC Corporation, Tokyo, Japan) to evaluate ML in DME across several experimental groups, in addition to a control group. The distal proximal box was contaminated with fresh human saliva to simulate clinical conditions. Under contamination-free conditions, DME performed with GIC Fuji IX GP showed significantly higher ML values (*p* < 0.05) compared with the groups restored with RMGIC (Fuji II LC and Equia Forte) as well as with the control group. However, under saliva contamination conditions, no statistically significant differences were observed among the evaluated groups (*p* > 0.05).

Lorca et al. [28] compared CC (Filtek™ Z250, 3M ESPE) placed in incremental layers of less than 2.5 mm thickness with a BFRC (Filtek™ Bulk Fill, 3M ESPE) applied using a single-increment technique of up to 5 mm thickness during DME. The authors reported no statistically significant differences in ML between BFRC (Filtek™ Bulk Fill) and CC (Filtek™ Z250) (*p* = 0.68). Nevertheless, a lower tendency toward ML was observed when using BFRC (28.6%) compared with the CC group (33.1%).

Koken et al. [12] evaluated the influence of the adhesive system on the cementation of direct restorations in samples with and without DME. For DME, a FRC (Gænial Universal Flo, GC Corporation, Japan) was used in combination with a universal adhesive (G-Premio Bond, GC Corporation, Japan) in all specimens. During the cementation procedure, two adhesive systems were compared: G-Premio Bond (GC Corporation, Japan) and OptiBond™ FL (Kerr Corporation, Orange, CA, USA). When G-Premio Bond was used for cementation, DME significantly increased ML (*p* = 0.00) compared with the control group, whereas no significant differences were observed when OptiBond FL was used (*p* > 0.05). Furthermore, in the absence of DME, G-Premio Bond showed lower ML than OptiBond FL.

Ghiorge et al. [35] evaluated ML in DME using the open sandwich technique, employing a compomer as the base material. The authors found that significantly lower ML was observed when the compomer (Dyract®, Dentsply Sirona, Konstanz, Germany) was placed at the gingival floor, but only when the restoration was subsequently completed with a CC (Zmack Comp, Zhermack SpA, Badia Polesine, Italy) and a total-etch adhesive system (Zmack® Bond Total Etch Adhesive, Zhermack SpA, Italy). In contrast, when GIC (Ketac™ Molar Easymix, 3M ESPE, St. Paul, MN, USA) was used at the gingival floor, the highest ML values were observed when the samples were restored with CC Zmack Comp, as well as with CC Filtek™ Z250 (3M ESPE, St. Paul, MN, USA) (*p* < 0.05). These findings were consistent with and supported by SEM evaluation.

Zavattini et al. [36] analyzed the clinical behavior of preheated composite and reported significantly higher ML when DME was performed using a FRC (Premise™ Flowable, Kerr Corporation, Orange, CA, USA) compared with preheated CC (Premise™ Dentin A3, Kerr Corporation, Orange, CA, USA). Notably, both groups employed the same three-step adhesive system (OptiBond™ FL, Kerr Corporation, Orange, CA, USA). Despite the use of the same adhesive protocol, the FRC group showed significantly greater ML than the preheated CC group (*p* < 0.05).

Raju et al. [37] demonstrated that DME performed with tricalcium silicate cement (Biodentine™, Septodont, Saint-Maur-des-Fossés, France) resulted in significantly lower ML compared with GIC (Fuji IX GP, GC Corporation, Tokyo, Japan) in both permanent teeth (*p* = 0.02) and primary teeth (*p* = 0.006). However, when GIC was used, it exhibited higher shear bond strength than the tricalcium silicate cement.

Similarly, Sawani et al. [29] performed DME using RMGIC (Vitrebond, 3M/ESPE, St. Paul, MN, USA) and FRC (Filtek 350 XT Flow, 3M/ESPE, St. Paul, MN, USA). The authors reported that restoration using the centripetal technique without DME resulted in significantly lower ML values compared with the centripetal closed sandwich technique with DME, which in turn showed lower ML than the centripetal open sandwich technique with DME (*p* < 0.05). Additionally, a trend toward lower ML was observed when FRC was used.

## Discussions

All 18 included studies were in vitro investigations with a cross-sectional experimental design. All studies applied random allocation, and four specified a single-blind design [9, 16, 28, 29]. Overall, the studies evaluated 945 extracted human posterior teeth that were sound, comparable, and free of caries, restorations, or cracks. These teeth were extracted for periodontal or orthodontic reasons with informed consent.

Class II cavities showed variations in their design, mainly with subgingival margins located 1–1.5 mm below the CEJ, except in three studies that positioned them 2 mm below the CEJ [11, 28, 31], while two studies did not specify the margin location [30, 37]. This variability may partially explain the heterogeneity observed in the results. Regarding the rehabilitation of the teeth after DME, direct restorations predominated, although four studies used indirect restorations (zirconia or lithium disilicate) [9, 12, 31] and one study employed a semidirect restoration using a composite resin inlay [28].

The aging and ML evaluation protocols were also heterogeneous, varying in the number and duration of thermocycling cycles, the use of nail varnish, the type of dye employed (methylene blue, ammoniacal silver nitrate, fuchsin, rhodamine B fluorescent solution, or ammonia), as well as the criteria used to assess ML. The primary visualization method was stereomicroscopy, although some studies additionally used digital microscopy, confocal scanning microscopy, micro-computed tomography, or SEM with different magnifications.

The methodological variations among the studies included the evaluation of different materials and restorative techniques. Consequently, although all studies aimed to assess ML in DME, the reported outcomes were often contrasting.

BF-FRC incorporate nanofillers that help maintain the mechanical properties of bulk-fill composites while preserving their low viscosity [38]. Furthermore, these materials show excellent adaptation to cavity margins [39], a high degree of conversion [40], and an improved capacity to relieve polymerization shrinkage stress [41]. Senol et al. [27] demonstrated that using Tetric N-Flow Bulk Fill, Ivoclar Vivadent in 2-mm increments resulted in significantly lower ML compared with 4-mm increments. Moreover, these thinner increments favored better marginal adaptation and a lower incidence of ML in SEM evaluation. This advantage may be explained by more efficient monomer conversion and reduced polymerization shrinkage. These findings support the clinical recommendation to limit the application of flowable resins to 2 mm increments in DME procedures, particularly in margins located on cementum, where marginal integrity is more susceptible to adhesion defects and polymerization contraction.

Similarly, Juloski et al. [9] reported that when comparing BF-FRC with FRC, the use of BF-FRC (Tetric EvoFlow® Bulk Fill, Ivoclar Vivadent) combined with a universal adhesive (Adhese® Universal, Ivoclar Vivadent) showed significantly lower ML values. These results suggest that this combination may optimize marginal sealing in DME procedures, potentially improving the clinical longevity of restorations. These findings are consistent with those reported by Lefever et al. [42], who also observed superior marginal adaptation to root dentin when BF-FRC was used in the DME technique. However, Juloski et al. also performed SEM analysis to evaluate the percentage of gap-free margins in areas with DME and did not find significant differences among the evaluated groups. This indicates that no statistically significant correlation was observed between ML scores and the marginal adaptation detected by SEM at DME sites.

On the other hand, Lorca et al. [28] reported that no statistically significant differences in ML were found between BFRC (Filtek™ Bulk Fill, 3M) and CC (Filtek™ Z250, 3M) when performing DME, although a lower tendency toward ML was observed with BFRC (28.6%) compared with the CC group (33.1%). Additionally, Ilgenstein et al. [43] and Zaruba et al. [44] indicated that BFRC composites exhibit greater resistance to deformation and lower thermal contraction during polymerization, demonstrating superior performance compared with materials with a lower elastic modulus, which may contribute to a reduction in ML when FRC are not used.

In contrast, Zhang et al. [31] reported that DME performed with a BF-FRC (Dentsply Sirona) did not show statistically significant differences compared with CC (Filtek™ Z350 XT, 3M) under the specific experimental conditions of the study In this investigation, ML did not appear to depend solely on the use of DME, but rather primarily on the depth of the gingival margin relative to the CEJ. The group without DME with margins located 1 mm below the CEJ exhibited the lowest ML, whereas higher ML values were observed when margins were located 2 mm below the CEJ, regardless of whether DME was performed. These findings suggest that marginal depth is a determining factor influencing sealing performance, potentially having a greater impact than the restorative technique itself.

Furthermore, the number of resin increments is an important factor directly affecting the integrity of the restorative margin. Roggendorf et al. [13] recommended the use of three layers or increments when performing DME, due to better adhesion performance in deep proximal dentin. Similarly, Frankenberger et al. [45] observed that three increments of 1 mm significantly improved marginal quality in dentin, and additionally reported that DME performed with BFRC achieved superior interfacial marginal quality compared with CC.

Patil et al. [18] emphasized that the outcomes are influenced not only by the material used for DME (CC or giomer-based FRC), but also by the restorative technique employed. Their results showed that the snowplow technique resulted in lower ML compared with the open sandwich technique and the oblique incremental technique.

Similarly, Sawani et al. [29] compared RMGIC (Vitrebond, 3M ESPE) with FRC (Filtek 350 XT Flow, 3M ESPE) for DME and reported that the centripetal technique without DME resulted in lower ML compared with cases in which DME was performed using the centripetal technique combined with closed sandwich and open sandwich techniques. These findings may be explained by the absence of the RMGIC interface, which could favor ML. Furthermore, higher ML was observed when DME was performed using the centripetal closed sandwich technique compared with the centripetal open sandwich technique.

Moreover, considering that restorative materials may be used in different ways, Zavattini et al. [36] reported significantly lower ML when using preheated CC (Premise™ Dentin A3, Kerr Corporation) compared with FRC (Premise™ Flowable, Kerr Corporation) while employing the same adhesive system. Therefore, further comparative studies involving preheated composites, FRC, and BF-FRC under homogeneous conditions are recommended to determine the potential effectiveness of preheated composite in DME procedures.

Likewise, Roider et al. [11] used dual-cure CC (Clearfil DC Core Plus, Kuraray Noritake) with a two-step self-etch adhesive (Clearfil SE Bond 2, Kuraray Noritake) for DME and observed greater ML when the gingival margin was located 2 mm below the CEJ compared with the non-DME group, where the margin ended immediately apical to the CEJ. However, although the DME group showed greater ML, the lack of standardization in margin depth prevents attributing this result exclusively to DME. The subgingival depth of the margin is a determining factor in marginal sealing quality and may therefore act as a confounding variable.

Bilgrami et al. [30] reported that the use of BF-FRC (SDR®, Dentsply Sirona) for DME demonstrated better adhesion capacity, showing minimal or no ML compared with RMGIC (Vitremer™, 3M ESPE) indicating greater compatibility between BF-FRC and the restorative materials evaluated. Furthermore, the use of the adhesive system (Single Bond™ Universal, 3M ESPE) improved marginal sealing regardless of the material used. However, the use of artificial saliva in this study represents only an approximation of clinical conditions and does not fully reproduce the characteristics of the oral cavity, which could influence the comparability of the results.

When applying the open sandwich technique, Feiz et al. [26] reported that using RMGIC (Ionoseal®, VOCO GmbH) for DME, or the absence of DME, resulted in greater ML compared with RMGIC (Fuji II LC, GC Corporation), BF-FRC (x-tra base®, VOCO GmbH), and (Grandio® Flow, VOCO GmbH). Therefore, this material appears to be less favorable for marginal adaptation when the open sandwich technique is employed. Despite technological advances in restorative materials, the results revealed that GIC exhibited behavior comparable to FRC in terms of ML, raising questions about the true magnitude of the improvement offered by FRC for this parameter.

Using the same technique, Gyanani et al. [32] reported that FRC giomer (Beautifil II Flow, Shofu Inc.) showed a greater tendency toward ML than RMGIC (Fuji II LC, GC Corporation) although the difference was not statistically significant. Similarly, Czarnecka et al. [46] suggested that RMGIC adhesion may hinder material compaction in confined areas and compromise marginal sealing.

However, Grubbs et al. [47] reported that DME performed with RMGIC presented fewer marginal defects after cyclic fatigue, which was attributed to the hygroscopic expansion of the material, increasing its volume and contributing to reduced ML. Nevertheless, this same characteristic may eventually lead to long-term hydrolytic degradation of the material.

Furthermore, Reddy et al. [33] demonstrated that GIC containing nano-hydroxyapatite (Nanowings, Pvt. Ltd.) showed lower ML compared with FRC (GrandioSO Heavy Flow®, VOCO GmbH) and GIC (GC Gold Label, GC Corporation). Despite showing less uniform marginal adaptation under SEM analysis, nano-hydroxyapatite GIC still presented lower ML.

Conversely, Çelik et al. [34] reported lower ML when DME was performed with RMGIC (Fuji II LC and Equia Forte®, GC Corporation) compared with GIC (Fuji IX GP, GC Corporation) but only in the absence of saliva contamination. In contrast, when saliva contamination was present, no statistically significant differences were observed between materials. However, these findings should be interpreted cautiously when extrapolated to clinical conditions. Even when DME is performed under absolute isolation, the restoration will eventually be exposed to the oral environment after removal of the rubber dam, facing saliva, thermal changes, masticatory load, and pH fluctuations. Therefore, the differences observed in vitro may not fully reflect real clinical behavior, where resistance to degradation and the ability to maintain long-term marginal sealing may be more relevant.

Raju et al. [37] demonstrated that tricalcium silicate (Biodentine™, Septodont) resulted in significantly lower ML compared with GIC (Fuji IX GP, GC Corporation). Nevertheless, GIC showed greater shear bond strength, attributed to the chemical interaction with dentin and micromechanical interlocking generated after conditioning. However, Vertolli et al. [48] reported no significant differences between GIC and RMGIC used for DME, observing satisfactory marginal integrity in a 5-year clinical follow-up, and also noted that DME contributed to reducing the incidence of ceramic fractures.

Additionally, Taori et al. [16] confirmed that when zirconomer (Zirconomer, Shofu Inc.) was used as the base material, there was a trend toward lower ML with the open sandwich technique, which was consistent with the observations obtained through SEM analysis.

In contrast to previous studies, Ghiorge et al. [35] reported lower ML when a compomer (Dyract®, Dentsply Sirona) was used as the base material for DME only when the restoration was subsequently completed with CC (Zmack Comp, Zhermack SpA) and a total-etch adhesive (Zmack® Bond Total Etch Adhesive, Zhermack S.p.A.). Conversely, when GIC (Ketac™ Molar Easymix, 3M ESPE) was used as the base, higher ML was observed regardless of the restorative composite used, a finding that was also confirmed through SEM analysis.

Although ML is closely related to the restorative material used for DME, the adhesive system also plays a crucial role in marginal integrity. The interaction between the adhesive and the restorative material influences seal formation, resistance to mechanical fatigue, and long-term stability, meaning that adhesive selection may significantly affect the clinical performance of the restoration [9]. In DME procedures, the hybrid layer is located in radicular dentin, as adhesion depends directly on the chemical interaction between monomers and calcium [49]. The 10-methacryloyloxydecyl dihydrogen phosphate (10-MDP) monomer present in universal adhesives is capable of forming additional chemical bonds by ionic interaction with hydroxyapatite calcium, producing a stable interfacial complex [50]. Furthermore, the hydrophobic nature of this structure protects the hybrid layer from degradation [51].

Consistent with these findings, Aljamhan et al. [25] reported that RMGIC showed the lowest ML among all groups. When a total-etch adhesive system was used, the lowest ML was observed with a combination of CC and FRC, whereas no significant differences were found between RMGIC and BFRC when a self-etch adhesive was used. However, the total-etch system showed higher ML across all groups, suggesting that adhesive type significantly influences marginal integrity in DME, with self-etch systems appearing more favorable. This may be explained by the enzymatic degradation and hydrolysis of collagen fibrils in the hybrid layer associated with total-etch systems [52]. In contrast, in self-etch adhesives, including universal adhesives used in self-etch mode, dentin demineralization and resin infiltration occur simultaneously [25]. Moreover, self-etch adhesive systems are believed to result in lower postoperative sensitivity and pain due to smear layer modification and greater sealing of dentinal tubules compared with total-etch systems [53].

In contrast, Ilgenstein et al. [43] and Theisen et al. [54] reported that the three-step adhesive (OptiBond™ FL, Kerr) showed no significant effect on ML, which is consistent with the findings of Zaruba et al. [44] using the three-step adhesive system (Syntac Primer, Syntac Adhesive, Heliobond, Ivoclar Vivadent).

Similarly, Koken et al. [12] highlighted the importance of the adhesive system selected during the cementation process, regardless of whether DME was performed. When G-Premio Bond (GC Corporation) was used during cementation in DME cases, ML increased compared with the control group. Conversely, when OptiBond™ FL (Kerr Corporation) was used, no significant differences were observed between groups. Furthermore, in the absence of DME, G-Premio Bond demonstrated lower ML than OptiBond FL. These results suggest that adhesive selection plays a decisive role in marginal sealing, particularly during the cementation phase.

This review presents several methodological limitations that should be considered when interpreting the findings. The most relevant limitation is the heterogeneity among the included studies, including differences in sample size, experimental design, restorative approaches, cavity designs, ML evaluation methods, DME materials, and adhesive systems, all of which may represent potential sources of bias. Consequently, there is a clear need for more standardized protocols that reduce methodological variability and improve the external validity of results. Nevertheless, despite these limitations, this review provides valuable information that may guide future in vitro and clinical research toward more rigorous and comparative approaches.

The absence of prospective protocol registration constitutes a methodological limitation; likewise, the conversion of the study from a narrative review to a systematic review during peer review may have introduced potential biases in the initial phases of study identification and selection. It should also be emphasized that although some studies have reported lower marginal ML with the centripetal technique, while others have shown favorable results for the snowplow technique, no direct comparative studies evaluating both techniques under equivalent methodological conditions are currently available. Therefore, it is not possible to establish a conclusive discussion supported by high-quality comparative evidence. Additionally, limited access to certain full-text articles may have influenced study selection and introduced a potential selection bias, as potentially relevant data were excluded for reasons unrelated to scientific quality. Therefore, clinical extrapolation remains limited, and the findings should primarily guide hypothesis generation rather than clinical decision-making. For this reason, clinical studies were excluded due to the methodological heterogeneity and inconsistent findings among in vitro studies, whose evidence is still insufficient for clinical application. However, in vitro studies remain an essential stage in research, providing the preliminary evidence required to initiate clinical investigations. Therefore, future comparative studies should evaluate the marginal performance of preheated CC, FRC, and BF-FRC using different restorative techniques, particularly focusing on long-term durability under simulated oral conditions. The absence of a meta-analysis constitutes a limitation; however, the methodological heterogeneity and variability in outcomes among the included studies justified the use of a qualitative synthesis.

Likewise, further investigations comparing all materials under standardized conditions, procedures, and techniques are necessary to obtain consistent results that can be reliably translated into clinical dental practice.

## Conclusions

Among the materials most commonly used for DME, composite resins stand out. The available evidence highlights the superiority of BF-FRC in reducing ML at the tooth–DME interface compared with other types of resin materials. Additionally, the use of RMGIC demonstrated superiority over conventional GIC, although not when compared with the different types of composite resins. Given the high variability of the reported results, these findings should be interpreted with caution. Furthermore, current evidence suggests that the use of self-etch adhesive systems results in reduced ML when performing DME. Finally, further comparative studies are recommended to determine which restorative technique provides the lowest ML. Although techniques such as the snowplow and centripetal techniques have shown favorable results, it is not currently possible to establish which technique is more effective due to the heterogeneity of the available studies and the limited number of investigations directly comparing them.

## Supplementary Material



## Data Availability

The datasets generated and analyzed during the current study are available from the corresponding author upon reasonable request.
