# Supplementary material for: Comparison of microleakage in restorative materials used for deep cervical margin elevation: a systematic review
Source: Biomater Investig Dent. 2026 Jul 1;13:46289. doi: 10.2340/biid.v13.46289 (PMC13332537; doi:10.2340/biid.v13.46289)
Supplement: Supplementary file 1 [file BIiD-13-46289-s1.pdf]

Supplementary material has been published as submitted. It has not
been copyedited or typeset by Biomaterial Investigations in Dentistry.

Table 1. PRISMA 2020 Checklist

Section/Topic	Item No	Checklist Item	Location of Item in the Report
**TITLE**			
Title	1	Identify the report as a systematic review	P 1
**ABSTRACT**			
Structured abstract	2	See the PRISMA 2020 for Abstracts checklist (Table 2).	P1-2
**INTRODUCTION**			
Rationale	3	Describe the rationale for the review in the context of existing knowledge.	P 3
Objectives	4	Provide an explicit statement of the objective(s) or question(s) addressed by the review.	P 3
**METHODS**			
Eligibility criteria	5	Specify the inclusion and exclusion criteria for the review and how studies were grouped for the synthesis.	P 4 y Table 1
Information sources	6	Specify all databases, registers, websites, organizations, reference lists, and other sources searched or consulted to identify studies. Indicate the date on which each source was last searched or consulted.	P 4
Search strategy	7	Present the full search strategies for all databases, registers, and websites, including any filters and limits used.	P 4-5 y Table 2
Selection process	8	Specify the methods used to determine whether a study met the inclusion criteria, including how many reviewers screened each record and each retrieved report, whether they worked independently, and, if applicable, details of automation tools used.	P 5 y Table 3
Data collection process	9	Specify the methods used to extract data from reports, including how many reviewers collected data from each report, whether they worked independently, processes for obtaining or confirming data from study investigators, and, if, applicable, details of automation tools used.	P 5

Section/Topic	Item No	Checklist Item	Location of Item in the Report
Data ítems	10a	List and define all outcomes for which data were sought. Specify whether results compatible with each outcome domain were sought (e.g., all measurement scales, time points, analyses) and, if not, the methods used to decide which results to collect.	
	10b	List and define all other variables for which data were sought (e.g., participant and intervention characteristics, funding sources). Describe any assumptions made about missing or unclear information.	
Risk of bias assessment in individual studies	11	Specify the methods used to assess risk of bias in the included studies, including details of the tool(s) used, how many reviewers assessed each study, whether they worked independently, and, if applicable, details of automation tools used.	P 6 y Table 5
Effect measures	12	Specify, for each outcome, the effect measures (e.g., risk ratio, mean difference) used in the synthesis or presentation of results.	P 5
Synthesis methods	13a	Describe the process used to decide which studies were eligible for each synthesis (e.g., tabulating study intervention characteristics and comparing them against planned groups for each synthesis (Item 5)).	P 5
	13b	Describe any methods required to prepare data for presentation or synthesis, such as handling missing summary statistics or data conversions.	P 5
	13c	Describe methods used to tabulate or visually display the results of individual studies and their synthesis.	P 5
	13d	Describe the methods used to synthesize results and justify their selection. If meta-analysis was performed, describe the models, methods used to identify statistical heterogeneity, and software used.	P 5
	13e	Describe any methods used to explore possible causes of heterogeneity among study results (e.g., subgroup analysis, meta-regressions).	-
	13f	Describe any sensitivity analyses conducted to assess the robustness of the synthesized results.	P 5
Reporting bias assessment	14	Describe methods used to assess risk of bias due to missing results in a synthesis (arising from reporting biases).	P 6

Section/Topic	Item No	Checklist Item	Location of Item in the Report
Certainty assessment	15	Describe methods used to assess certainty (or confidence) in the body of evidence for each outcome.	P 6
**RESULTS**			
Study selection	16a	Describe results of the search and selection process, from the number of records identified to the number of studies included, ideally using a flow diagram (see Figure 1).	P 6 y Figure 2
	16b	Cite studies that appeared to meet inclusion criteria but were excluded, and explain why they were excluded.	
Study characteristics	17	Cite each included study and present its characteristics.	P 8, 9 y 10 y Table 4
Risk of bias in studies	18	Present risk of bias assessments for each included study.	P 7 y Table 6
Results of individual studies	19	For all outcomes and for each study, present: (a) summary statistics for each group (if applicable) and (b) effect estimates and their precision (e.g., confidence or credibility intervals), ideally using structured tables or plots.	P 8, 9 y 10
Results of synthesis	20a	For each synthesis, briefly summarize the characteristics and risk of bias among contributing studies.	P 7
	20b	Present the results of all statistical syntheses conducted. If meta-analysis was performed, present the summary estimate and its precision (e.g., confidence or credibility interval) and measures of statistical heterogeneity. If groups were compared, describe the direction of effect.	No metanalysis
	20c	Present results of all investigations into possible causes of heterogeneity among study results.	P 8, 9 y 10
	20d	Present the results of all sensitivity analyses conducted to assess robustness of synthesized results.	P 8, 9 y 10
Reporting biases	21	Present assessments of risk of bias due to missing results (arising from reporting bias) for each synthesis assessed.	-

Section/Topic	Item No	Checklist Item	Location of Item in the Report
Certainty of evidence	22	Present assessment of certainty (or confidence) in the body of evidence for each outcome evaluated.	-
**DISCUSSION**			
Discussion	23a	Provide a general interpretation of the results in the context of other evidence	P 15-19
	23b	Discuss the limitations of the evidence included in the review.	P 20
	23c	Discuss the limitations of the review processes used.	P 20
	23d	Discuss implications of the results for practice, policy, and future research.	P 20
**OTHER INFORMATION**			
Registration and protocol	24a	Provide registration information for the review, including name and registration number, or state that no protocol was prepared.	P 3
	24b	Indicate where the protocol can be accessed, or state that the review was not registered.	P 3
	24c	Describe and explain any amendments to the information provided in the registration or protocol.	P 3
Funding	25	Describe sources of financial or non-financial support for the review and the role of funders or sponsors.	P 21
Competing interests	26	Declare conflicts of interest of review authors.	P 21
Availability of data, code, and other materials	27	Specify which of the following are publicly available and where they can be found: data extraction forms, extracted data from included studies, data used for analyses, analytic code, and any other materials used in the review.	P 21
